# CD133 induces tumour-initiating properties in HEK293 cells

**DOI:** 10.1007/s13277-012-0568-z

**Published:** 2012-11-13

**Authors:** Martin Canis, Axel Lechner, Brigitte Mack, Pamela Zengel, Rüdiger Paul Laubender, Udo Koehler, Vigo Heissmeyer, Olivier Gires

**Affiliations:** 1 Department of Otorhinolaryngology, Head and Neck Surgery, Grosshadern Medical Centre, Ludwig–Maximilians University of Munich, Marchioninistr. 15, 81377 Munich, Germany; 2 Department of Otorhinolaryngology, Head and Neck Surgery, University of Göttingen, Robert-Koch-Str. 40, 37075 Göttingen, Germany; 3 Institute of Medical Informatics, Biometry, and Epidemiology, Ludwig–Maximilians University of Munich, Munich, Germany; 4 Medizinisch Genetisches Zentrum (MGZ), Bayerstraße 3-5, 80335 Munich, Germany; 5 Helmholtz Zentrum München, German Research Center for Environmental Health, Marchioninistr. 25, 81377 Munich, Germany; 6 Head and Neck Research Department, Ludwig–Maximilians University of Munich, Marchioninistr. 15, 81377 Munich, Germany

**Keywords:** CD133, HEK293, Tumourigenic potential, Mouse model

## Abstract

The pentaspan protein CD133 (Prominin-1) is part of the signature of tumour-initiating cells for various cancer entities. The aim of the present study was to investigate the impact of ectopic CD133 expression on tumourigenic properties of otherwise CD133-negative, non-tumourigenic cells in vitro and in vivo. CD133 was stably transfected into human embryonic kidney 293 (HEK293) which was then sorted for the expression of CD133. The effects of CD133 on cell proliferation were assessed upon standard cell counting of sorted cells at various time points. Severe combined immunodeficient (SCID) mice (*n* = 30) were injected with HEK293 CD133^high^ and CD133^low^ transfectants (5 × 10^3^, 1 × 10^5^, or 5 × 10^6^ cells per injection). The expression of CD133, Ki67, CD44s, CD44v6, and EpCAM was analysed upon immunohistochemical staining of cryosections with specific antibodies. In vitro, ectopic expression of CD133 did influence neither cell proliferation nor cell cycle distribution of otherwise CD133-negative HEK293 cells. However, CD133^high^ cells generated tumours in vivo in SCID mice with at least 1,000-fold increased frequency compared to CD133^low^ cells. Tumour load was also significantly increased in CD133^high^ cells as compared to those tumours formed by high numbers of CD133^low^ cells. Immunohistochemistry stainings disclosed no changes in Ki67, CD44s, CD44v6, or EpCAM once tumours were formed by either cell type. CD133 induces tumour-initiating properties in HEK293 cells in vivo and is potentially involved in the regulation of tumourigenicity. Future research will aim at the elucidation of molecular mechanisms of CD133-induced tumourigenicity.

## Introduction

Accumulating data demonstrate that malignant tumours are structured hierarchically, and their formation is driven by a small sub-population of tumour-initiating cells (TICs). These cells show self-renewal capacity and may be responsible for tumour progression and metastasis [1]. TICs, also termed as cancer-stem cells, were first identified in human acute myeloid leukaemia and represented cells expressing the cell surface markers CD34^high^ and CD38^low^ [2, 3]. Similar to the haematopoetic system, epithelial linings undergo continuous turnover and are hierarchically organised according to a stem cell system [4]. In 2004, CD133^high^ cells were isolated from the human brain tumours, which showed tumour-initiating capacity and recapitulated the original phenotype of the tumour of origin after serial transplantation in vivo [5]. CD 133 is a ∼120-kDa glycoprotein with an N-terminal extracellular domain, two large extracellular loops, and an intracellular C-terminus [6]. In vitro experiments revealed that the expression of CD133 in cell lines is associated with enhanced clonogenicity and tumourigenicity [7, 8]. CD133 thus obtained great attention, owing to its high expression in the form of a hyper-glycosylated variant in TICs of various origins [1, 9, 10]. Very recently, a role for CD133 and the Src kinase in the regulation of tumour initiating properties and the transition from an epithelial to a mesenchymal phenotype of head and neck carcinoma cells has been demonstrated [11]. Beside CD133, markers such as CD24, EpCAM, CD166, Lgr5, CD47, and ALDH have been discussed and serve for the selection of tumour-initiating cells [12]. The aim of the present study was to investigate the impact of ectopic CD133 expression on tumourigenic properties of otherwise CD133-negative, non-tumourigenic cells in vitro and in vivo. De novo expression of CD133 in human embryonic kidney 293 (HEK293) cells conferred tumour-initiating capacity to these otherwise CD133-negative cells, strongly suggesting that CD133 actively contributes to the TIC phenotype of malignant cells.

## Materials and methods

### Cell lines and cell counting

HEK293 cells [13] and CaCo-2 colon carcinoma cells were purchased from ATCC. CaCo-2 cells express CD133 endogenously and therefore served as a positive control. HEK293 transfectants were generated upon magnet-assisted transfection (MaTra, Iba, Göttingen, Germany) of the pCR3.1-uni vector, in which the cDNA for CD133 was introduced by conventional cloning. The selection of stable transfectants was achieved with standard DMEM medium supplemented with G418 (Calbiochem, Merck GmbH, Schwalbach, Germany). Stable transfectants were sorted for their CD133 expression profile in a FACSAria II device (BD Biosciences, Heidelberg, Germany). Sorted cells were plated in 35-mm dishes at different densities. Cell numbers were assessed at different time points upon trypan blue exclusion assay in Neubauer counting chambers.

### Immunoblot and PNGase F treatment

Cells were lysed in 50 μl lysis buffer (1 % Triton in TBS), and protein amounts were assessed with the BCA™ Protein Assay Kit (Pierce, Thermo Scientific, Rockford, IL, USA). Lysates from CD133^high^ and CaCo cells were treated with PNGase F to deglycosylate proteins. PNGase F treatment (New England Biolabs, P0704S) of cell supernatants was conducted as recommended by the manufacturer.

Protein lysate (50 μg) was mixed with SDS-PAGE loading buffer (25 mM TrisHCl, pH 7, 5 % glycerin, 1 % SDS, 2 % beta-mercaptoethanol, bromphenol blue). The proteins were separated by SDS-PAGE, transferred onto PVDF membranes (Millipore, Bedford, MA, USA), and detected using specific antibodies in combination with horseradish peroxidase-conjugated secondary antibodies and the enhanced chemiluminescence reagent (Amersham Biosciences, Freiburg, Germany). Antibodies used are AC133 (Miltenyi, Bergisch Gladbach, Germany), CD133 (Cell Signalling, Danvers, MA, USA), AC133 and CD133 which are two different clones for the same molecule, Ki67 (Dako, Hamburg, Germany), splice variant of CD44s (BD Pharmingen, Heidelberg, Germany), CD44v6 (Novocastra, Newcastle upon Tyne, UK), EpCAM (HO.3) [14] (kind gift of Dr. P. Ruf, Trion Research, Munich Germany), and actin (Santa Cruz Biotechnology Inc., Santa Cruz, CA, USA).

### Flow cytometry

Cells were stained with a 1:50 dilution of an anti-CD133 antibody (AC133, Miltenyi) or an isotype control (IgG1, Dako) for 15 min on ice, washed three times in PBS supplemented with 3 % of foetal calf serum (FCS), and stained with fluorescein isothiocyanate-conjugated secondary antibody. Measurement of cell surface expression of CD133 was performed in a FACSCalibur device (BD Pharmingen).

### Semi-quantitative RT-PCR

Total RNA from cell lines was isolated using the High Pure RNA Isolation Kit (Macherey & Nagel, Dueren, Germany), and the cDNA was generated using the reverse transcription system (Promega, Madison, WI, USA) according to the manufacturer's instructions. Semi-quantitative PCR analysis for the expression of CD133 and GAPDH (95 °C for 30 s, annealing for 30 s, 72 °C for 30 s) was conducted with specific primers: for CD133 (194 bp), 5′-tggggctgctgtttattattct-3′ and 5′-tgccacaaaaccatagaagatg-3′, while for GAPDH (258 bp), FW: 5′-tgtcgctgttgaagtcagaggaga-3′ and BW: 5′-agaacatcatccctgcctctactg-3′.

### Xenotransplantation model

Six-week-old severe combined immunodeficient (SCID) male mice (*n* = 30) were injected with HEK293 CD133^high^ and CD133^low^ transfectants subcutaneously into the right and left flanks of the same mouse, respectively (5 × 10^3^, 1 × 10^5^, or 5 × 10^6^ cells per injection in 100 μl medium and 100 μl Matrigel, BD Pharmingen). All experiments were performed according to Bavarian state regulations for animal experimentation and were approved by the responsible authorities, the District Government of Upper Bavaria (Animal licence no.: 55.2-1-54-2531-101-07). After 21–36 days, the mice were killed in a CO_2_ gas environment. Subsequently, all tumours were dissected and removed, weighed, and immediately embedded in Tissue-Tek before cryopreservation in liquid nitrogen and storage at −20 °C. Immunohistochemistry of the mouse samples was performed as described for human materials. Human antigens were detected with CD133 pure (Miltenyi, dilution 1:100), CD44v6 (Novocastra, dilution 1:300), CD44s (BD Pharmingen, dilution 1:4,000), and Ki67 (Dako, dilution 1:800) antibodies. For isotype control, mouse IgG1 (Dako) antibody was used, while mouse IgG2b antibody (Sigma) served as negative control for CD44s. Immunodetection of primary antibodies was carried out using biotinylated anti-mouse antibody coupled with peroxidase-labelled avidin–biotin complex (Vector Lab. Inc., Burlingame, CA, USA).

## Results

### CD133 expression in vitro in HEK293 cells

**Fig. 1:**
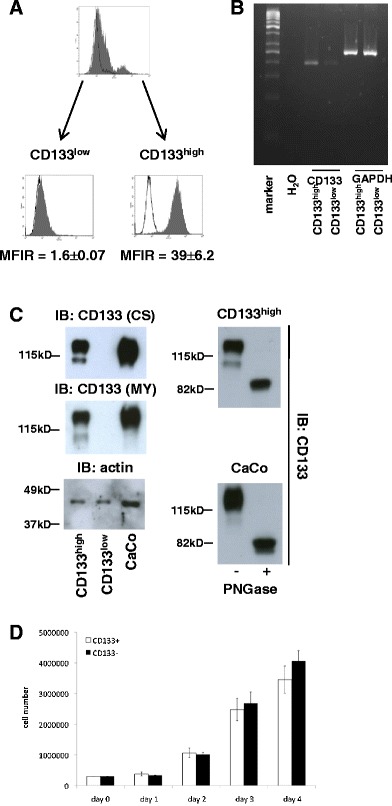
CD133 expression in HEK293 cells. **a** HEK293 cells were stably transfected with an expression plasmid for CD133. Flow cytometry analysis revealed mixed populations of CD113^low^ and CD133^high^ cells within single stable transfectants. Cells were sorted to obtain pure populations with mean fluorescence intensities ratios of CD133^low^ = 1.6 ± 0.07 and CD133^high^ = 39 ± 6.2. **b** CD133 mRNA RNA levels were assessed by standard RT-PCR in CD113^low^ and CD133^high^ cells. GAPDH mRNA levels served as control. **c** CD133 protein levels were assessed upon immunoblotting with two independent antibodies from two different clones for the same molecule (*CS* Cell System, *MY* Miltenyi). Colon carcinoma cells CaCo, which endogeneously express CD133, were assessed similarly as control. Actin was used as a loading control (*left panels*). Lysates from CD133^high^ and CaCo cells were treated with PNGase to deglycosylate proteins. Thereafter, lysates of untreated cells and treated lysates were separated in a 10 % SDS-PAGE and CD133 detected with specific antibody (*MY*, *right panels*). **d** Equal cell numbers (3 × 10^5^ cells) of HEK293 CD113^low^ and CD133^high^ cells were plated, and the cell numbers were assessed at the indicated time points. Shown are the mean and standard deviations of the three independent experiments

In order to address a putative role of CD133 in the regulation of the oncogenic potential of cells, HEK293 cells were stably transfected with an expression plasmid for CD133. HEK293 clones used under the experimental conditions applied herein possess only very low tumourigenic capacity and hardly generate tumours within a time span of 3 weeks [15]. Bulk cultures of stable transfectants were analysed for the cell surface expression of CD133 upon flow cytometry with specific antibodies. An example of one bulk culture is shown in Fig. 1a. Two spontaneously occurring sub-populations were observed which differed significantly in their expression profile of CD133. CD133^low^ and CD133^high^ cells were separated upon FACS sorting before every xenotransplantation, yielding two sub-populations with CD133 average mean fluorescence intensity ratios (CD133/controls) of 1.6 ± 0.07 and 39 ± 6.2, respectively (Fig. 1a, lower panels). Expression levels of CD133 mRNA and protein were additionally assessed upon RT-PCR and immunoblotting of the resulting cell populations (Fig. 1b, c). Even though CD133 expression was below the detection limit in CD133^low^ cells in immunoblot experiments, single cells were positive for CD133 as observed in the immunocytochemical stainings (data not shown). In CD133^high^ cells, CD133 protein expression was comparable to the endogenous levels seen in colon carcinoma cell line CaCo-2 independently of the primary anti-CD133 antibody used for detection (Fig. 1c). Ectopically expressed CD133 migrated in SDS-PAGE as two protein bands with an apparent molecular weight of 130 and 110 kDa (Fig. 1c). After the treatment of the cell lysates with glycosidase, CD133 derived from HEK293, CD133^high^, and from CaCo-2 cells shifted to a protein with an apparent molecular weight of approximately 83 kDa (Fig. 1c, right panels), thus suggesting that higher molecular weight species of CD133 resulted from post-translational glycosylation and represent closely the variant preferentially expressed in TICs [9]. Next, the proliferation capacity of both cell clones was assessed over a time period of 4 days. CD133^low^ and CD133^high^ cells were plated at equal cell numbers and counted daily. Both cell cultures grew exponentially, with CD133^low^ cells displaying minimally increased cell numbers as compared to CD133^high^ cells; however, there was no significant difference (Fig. 1d). These findings were corroborated by analyses of cell cycle distributions of both cell sub-populations. After re-addition of serum factors to serum-starved CD133^low^ and CD133^high^ cells (0.1 % FCS), CD133^low^ cells displayed slightly enhanced percentages of cells in S phase compared to CD133^high^ cells (data not shown).

### Tumour-initiation capacity of HEK293 cell clones in vivo

**Fig. 2:**
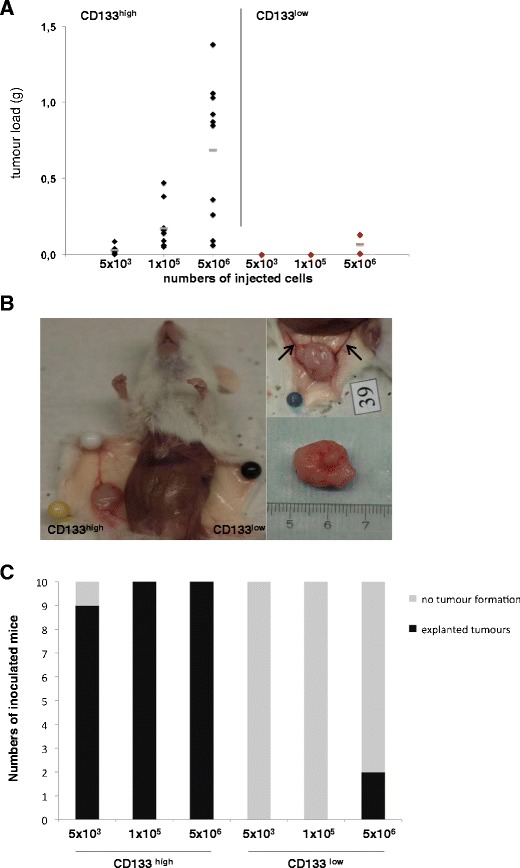
Measurement of the tumourigenic potential of CD113^low^ and CD133^high^ cells in vivo. **a** SCID mice were inoculated subcutaneously with increased amounts of CD113^low^ and CD133^high^ cells in the left and right flank, respectively. After 21 to 36 days, the animals were killed, and the tumour load in grams was monitored. The results shown represent two experiments with five mice each (*n* = 10). *Black and red diamonds* give the tumorload in regard of numbers of injected cells. **b** Tumours generated by CD113^low^ and CD133^high^ cells are exemplified before (*left* and *upper right images*) and after preparation (*lower right image*). The blood vessels supplying the tumours are marked with *arrows* in the *upper right panel*. **c** Tumour formation frequency of CD133^low^ and CD133^high^ HEK293 cells. SCID mice (*n* = 10 in two independent experiments) have been inoculated with the indicated cell numbers and tumour formation assessed over a time period of 21–36 days. Shown are frequencies of tumour formation: *grey bars* represent no formation of tumours, while *black bars* represent tumour formation independently of the tumour size and weight

We next tested the impact of CD133 expression on the ability of HEK293 cells to form tumours in vivo in immunocompromised SCID mice. Increasing numbers of CD133^low^ and CD133^high^ cells (5 × 10^3^, 1 × 10^5^, and 5 × 10^6^ cells per injection) were injected with Matrigel (1:1) in the left and right flank of the same SCID mouse (*n* = 10 per cell numbers). Tumour formation was recorded as the weight of resulting tumours at the site of injection. Tumours were allowed to generate within a time period of 21–36 days. CD133^low^ cells generated very small tumours in two out of the 30 injections (7 %) and only at the highest concentrations of 5 × 10^6^ cells per injection (Fig. 2a, c). CD133^high^ cells generated tumours in 29 out of the 30 injections (97 %) (Fig. 2c) and displayed a dose dependency with respect to the tumour mass (Fig. 2a). At concentrations of 5 × 10^3^ cells, CD133^high^ cells grew to tumours of sizes comparable to those generated by 5 × 10^6^ CD133^low^ cells (Fig. 2a). From these results, we deduced that CD133^high^ cells are leastwise a 1,000-fold enriched in tumourigenic cells compared with their CD133^low^ counterparts. At highest concentrations of 5 × 10^6^ CD133^high^ cells, tumours had maximal weights of 1.4 g, representing up to 7 % of the total body weight of the animal. Typically, tumours originating from the CD133^high^ cells were connected to blood vessels, which were almost entirely missing on the opposite side, which had been inoculated with CD133^low^ cells (Fig. 2b, arrows). Accordingly, CD133^high^ tumour specimens were well supplied with blood (see example in Fig. 2b, right panels).

**Fig. 3:**
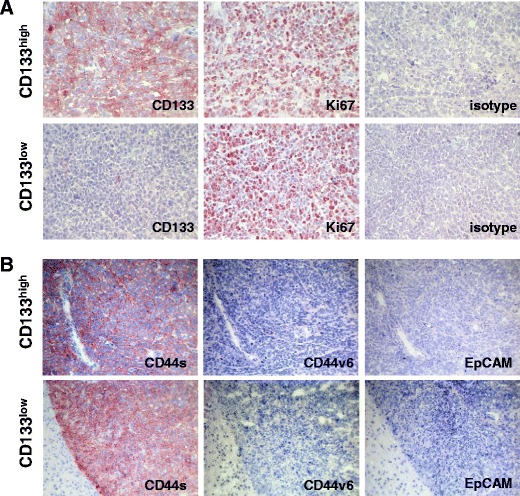
Immunohistochemical analysis of CD133, Ki67, CD44s, CD44v6, and EpCAM in HEK293-derived tumours. **a** Sections of cryopreserved samples of tumours derived from CD113^low^ and CD133^high^ cells inoculated in SCID mice were stained with CD133- and Ki67-specific antibodies and with an isotype-matched antibody for CD133 as a control. **b** Same as in **a**, but staining occurred with CD44s-, CD44v6-, and EpCAM-specific antibodies. Shown are representative micrograph sections from CD113^low^ and CD133^high^ cell-derived tumours

Tumour samples of both CD133^high^ and CD133^low^ cells were cryopreserved, processed to sections, and stained with antibodies specific for CD133, Ki67 as a marker for proliferation, and an isotype control antibody. CD133^high^-derived tumours (*n* = 29) were characterised by a homogeneous and high CD133 expression and by a high proliferation rate (exemplified in Fig. 3a, upper panels). In CD133^low^-derived tumours (*n* = 2), only single cells expressed CD133, reflecting the original expression pattern of CD133 determined in vitro, while proliferation was comparable to CD133^high^-derived samples. Additional transmembrane proteins such as CD44 and EpCAM have been associated with tumour-initiating frequency and are abundantly expressed in TICs of numerous tumour entities [1, 16]. In order to assess the potential differences in these molecules, CD133^high^- and CD133^low^-derived tumours were stained for the standard CD44s, CD44v6, and for EpCAM. Both tumour samples similarly expressed CD44s and did not display any reactivity for CD44v6 and EpCAM (Fig. 3b). Hence, differences in tumour formation are not due to the variations in the expression of CD44 or EpCAM.

## Discussion

The actual function of CD133 is just emerging, and until recently, an implication in the determination of haematopoietic stem cells (HSCs) and neuroepithelial progenitors was the only known role [17]. Most recent publications reported on the association of CD133 with Src kinase [18], which is instrumental in tumour initiation and during transition from an epithelial to a mesenchymal phenotype (EMT) [11]. In the present study, we demonstrate a potential for CD133 to induce a tumour-initiating phenotype in cells endowed with very low tumourigenicity. Tumour seeding frequency in CD133^high^ HEK293 cells was leastwise 1,000-fold higher than in CD133^low^ counterparts, although the cellular and morphologic features of these cells were indistinguishable in vitro. Especially, no traits of EMT were observed in these cells, including unchanged expression levels of typical EMT markers such as vimentin, N-cadherin, or smooth muscle actin (data not shown). This lack of difference between CD133^high^ and CD133^low^ cells in vitro is perfectly in accordance with previous reports on the knock-down of CD133 in colon carcinoma CaCo-2 cells, which was likewise without any measurable outcome in vitro [19, 20]. From their results, Horst et al. [19, 20] deduced a lack of function for CD133 in the regulation or induction of a TIC phenotype in vitro. The impact of CD133 on the tumour seeding capacity however suggests that it is important for the survival and/or regulation of tumour-seeding cells in the in vivo context. For example, anchorage-independent growth and/or communication with the microenvironment could account for differences between results from in vitro and in vivo studies. An involvement of CD133 in the cell-to-cell communication of HSCs and mesenchymal stem cells (MSCs) has been reported [21]. Migratory HSCs display a polarised localisation of CD133 at a rear part of the cell, a structure termed uropod, which is uplifted and does not contact the layer of MSCs beneath. Upon contact of the uropod with MSCs, HSCs become more sessile and cease migration [21]. Potential ligands of CD133 on MSCs or other cell types of the microenvironment have as yet not been explored, although they appear of great interest for the understanding of CD133's role in the communication of stem cells with their niche. Similar to HSCs, CD133 might serve tumourigenic cells as a means of contact and communication with the microenvironment in vivo, thus providing them with the capacity to interact and engraft. Accordingly, CD133^+^/CD24^+^ marked clonogenic TICs derived from single colon carcinoma cells in spheroid cultures or limiting dilutions [22]. CD133^+^/CD24^+^ cells possessed a multilineage differentiation potential, both in vitro and in vivo, while CD133^−^ cells did not. Interestingly, stromal cells in tumours generated upon xenotransplantation of CD133^+^/CD24^+^ cells were derived from the host and did not originate from CD133^+^/CD24^+^ tumour cells, hence indicating that CD133 might also be required for the proper communication of tumour cells and cells of the host's microenvironment [22].

In summary, CD133 has the potential to induce a tumour-initiating phenotype in low tumourigenic HEK293 cells in vivo. Since these results are descriptive only, future research will aim at the elucidation of molecular mechanisms of CD133-induced tumourigenicity, its putative role in the formation of metastases, which could be linked to interactions with Src kinase, and potentially pinpoint novel therapeutic intervention options.
